# Genetically Determined MBL Deficiency Is Associated with Protection against Chronic Cardiomyopathy in Chagas Disease

**DOI:** 10.1371/journal.pntd.0004257

**Published:** 2016-01-08

**Authors:** Paola Rosa Luz, Márcia I. Miyazaki, Nelson Chiminacio Neto, Marcela C. Padeski, Ana Cláudia M. Barros, Angelica B. W. Boldt, Iara J. Messias-Reason

**Affiliations:** 1 Laboratório de Imunopatologia Molecular–Departamento de Patologia Médica, Hospital de Clínicas, Universidade Federal do Paraná, Curitiba, Brasil; 2 Ambulatório de Atenção ao Paciente Chagásico—Hospital de Clínicas, Universidade Federal do Paraná, Curitiba, Brasil; 3 Serviço de Ecocardiografia—Hospital de Clínicas, Universidade Federal do Paraná, Curitiba, Brasil; 4 Laboratório de Genética Molecular Humana–Departamento de Genética, Universidade Federal do Paraná, Curitiba, Brasil; Albert Einstein College of Medicine, UNITED STATES

## Abstract

Chagas disease (CD) is caused by *Trypanosoma cruzi*, whose sugar moieties are recognized by mannan binding lectin (MBL), a soluble pattern-recognition molecule that activates the lectin pathway of complement. MBL levels and protein activity are affected by polymorphisms in the *MBL2* gene. We sequenced the *MBL2* promoter and exon 1 in 196 chronic CD patients and 202 controls. The MBL2*C allele, which causes MBL deficiency, was associated with protection against CD (P = 0.007, OR = 0.32). Compared with controls, genotypes with this allele were completely absent in patients with the cardiac form of the disease (P = 0.003). Furthermore, cardiac patients with genotypes causing MBL deficiency presented less heart damage (P = 0.003, OR = 0.23), compared with cardiac patients having the *XA* haplotype causing low MBL levels, but fully capable of activating complement (P = 0.005, OR = 7.07). Among the patients, those with alleles causing MBL deficiency presented lower levels of cytokines and chemokines possibly implicated in symptom development (IL9, p = 0.013; PDGFB, p = 0.036 and RANTES, p = 0.031). These findings suggest a protective effect of genetically determined MBL deficiency against the development and progression of chronic CD cardiomyopathy.

## Author Summary

Chagas disease is considered an important neglected tropical disease, affecting approximately ten million people in Latin America. Although most infected individuals remain asymptomatic, one third of patients develop a chronic heart disease, with progressive inflammation, increase of myocardium, arrhythmia, cardiac insufficiency and heart failure. To date, there is no available marker to indicate the progression neither to determinate the severity of heart damage. Mannan binding lectin (MBL) is an important protein of the immune system able to recognize specific regions on the microorganism surfaces (including *Trypanosoma cruzi*, the causal agent of Chagas disease) which activate the complement system, a crucial mechanism of the effector immunity. MBL levels and protein activity are affected by genetic differences, named polymorphisms, in the *MBL2* gene. This is the first Brazilian study with *MBL2* polymorphisms in chronic Chagas disease. We sequenced two regions of *MBL2* gene in 196 patients and 202 controls. We found that a polymorphism associated with deficient complement activation protects against Chagas disease and patients with deficiency-associated genotypes presented less echocardiographic alterations. Among the patients, those with alleles causing MBL deficiency presented lower levels of cytokines and chemokines possibly implicated in symptom development (IL9, p = 0.013; PDGFB, p = 0.036 and RANTES, p = 0.031). These findings lead us to suggest that genetically determined MBL deficiency plays a protective role against the development and progression of chronic Chagas disease.

## Introduction

Chagas disease (CD) is considered the most important neglected tropical disease worldwide, affecting approximately ten million people in Latin America [1,2,3]. The disease is caused by *Trypanosoma cruzi*, a flagellated protozoan parasite transmitted to humans mainly by blood-sucking triatomine bugs or by blood transfusion [4]. Approximately 50% of the individuals infected by *T*. *cruzi* remain in the indeterminate or asymptomatic clinical form of CD for their whole lives. Although asymptomatic patients present in general a good prognosis, each year about 2–5% of them progress to symptomatic forms of the disease, developing cardiac, digestive and/or neurological clinical manifestations [5]. About 30–40% of the patients develop chronic chagasic cardiomyopathy (CCC), characterized by progressive and multifocal inflammation, fibrosis and subsequent cardiac insufficiency (CI). In advanced stages, there is a marked increase in the heart in an attempt to compensate loss of function, with thromboembolic events, important arrhythmias and heart failure. Sudden death is a constant risk at any clinical stage, occurring in one to two thirds of patients who die due to CD. Although most of these patients presented prior CI, about one third to one fifth of sudden deaths occur in asymptomatic CD patients [6,7].

To date, there is no available marker which is able to indicate the progression from asymptomatic to symptomatic CD, neither to indicate the severity of the disease. Though predisposition to CD and progression to the different clinical forms are certainly modulated by several factors involved in the interaction of *T*. *cruzi* with the host [8].

The complement system is considered one of the major mediators of innate host defense, playing an important role in the control of experimental *T*. *cruzi* infection [9,10], as well as in clinic evolution of CD [11,12]. The activation of complement can occur by three different pathways. One of them is the lectin pathway, initiated through the recognition of sugar motifs on the pathogen’s surface by MBL (mannan-biding lectin), the collecting COLEC11 or ficolins. MBL is a soluble pattern-recognition protein (PRP) of the innate immunity which binds to specific pathogen-associated molecular patterns (PAMP) on microorganism surfaces, leading to complement activation. Additionally, MBL is able to promote inflammation, removal of apoptotic cells and opsonophagocytosis independently of complement [13,14].

It has been demonstrated that complement activation is critical for the control of CD and its depletion was associated with high parasitemia and early death in an experimental study using a mice model [9]. Moreover, there is much evidence about the involvement of complement in *T*. *cruzi* infection [15,16] as well as its important role in the clinical outcome of CD [12,17,18]. Besides its beneficial effect in the immune response, complement is known to play a significant role in various immune-mediated diseases. Increased concentrations of MBL were suggested to lead to an excessive activation of complement, with consequent exacerbation of inflammatory response, promoting tissue damage [19,20,21,22,23,24].

The human MBL gene (*MBL2*) is located on chromosome 10q11.2-q21 and contains five exons. Aminoacid substitutions caused by three single nucleotide polymorphisms (SNPs) located at codons 52 (Arg52Cys, allele *D*), 54 (Gly54Asp, allele *B*) and 57 (Gly57Glu, allele *C*) in exon 1 disrupt the collagenous tail of the protein and result in failure of production of fully functional multimeric protein, lowering MBL serum levels (*B*, *C* and *D* are referred together as “*O*” alleles). Heterozygous individuals for these mutations show substantial decrease in the concentration of circulating MBL, while homozygotes have almost undetectable serum MBL (less than 100 ng/ml), which characterizes deficiency of the protein. Aberrant MBL molecules do not bind effectively to PAMPs neither promote the activation of the lectin pathway. However, they may work as opsonins or mediate cellular cytotoxicity [25]. In addition, SNPs in the promoter and 5’ untranslated regions of the *MBL2* gene (*H/L*, *X/Y*, *P/Q* at positions -221, -550 and +4, respectively) are also known to affect the serum concentration of protein [26,27]. Three of the most common *MBL2* haplotypes—*HYPA*, *LYQA* and *LYPA*—are associated with increased expression of circulating MBL, whereas *LXPA*, *HYPD*, *LYPB* and *LYQC*—are associated with deficiency of this protein [28].

In a previous report we showed that high MBL levels were associated with the presence of echocardiographic alterations and cardiac insufficiency in patients with chronic CD from Brazil [29]. In this study, we extended the investigation in the same cohort of patients, in order to find out if the different protein levels result from *MBL2* polymorphisms or from the disease course. Given the important role of MBL in innate immunity and in the association of *MBL2* functional polymorphisms with different chronic, infectious, inflammatory and auto-immune diseases [27,30], *MBL2* polymorphisms seem to be good candidate markers for susceptibility and clinical progression of CD.

## Materials and Methods

### Subjects and samples

We investigated 196 chronic CD patients (59.2% female; average age 57.3 years (34–90); 74.5% Euro-, 20.4% Afro-Brazilian, 0.5% Asian, 5.0% Amerindian) from the Chagas Disease Ambulatory of the Clinical Hospital of the Federal University of Paraná (HC-UFPR). CD diagnosis was given by serological and clinical examinations. The clinical history of the patients was obtained from medical records and interviews, using a standard questionnaire. For three patients, the clinical form of CD was undefined at the time of sampling. Patients younger than 18 years-old, or that present history of blood transfusion, recent infections and suspected non-chagasic cardiomyopathy (such as hypertensive cardiomyopathy) were excluded. Detailed demographic and clinical characteristics of the specific CD forms are given in Table 1. A group of 202 unrelated adult individuals with negative Chagas (anti-*Trypanosoma cruzi*) serology were used as controls (56.9% female, average age 49.3 years (19–79), 74.3% Euro-, 20.8% Afro-Brazilian, 0.5% Asian, 4.5% Amerindian). Ethnic background of patients and controls was determined as previously described [31]. The project was approved by the ethics committee of Hospital de Clínicas, Universidade Federal do Paraná (CEP/HC-UFPR n.1457.122/2007-06).

**Table 1:** Demographic data and clinical parameters of Chagas patients.

		Chagas Clinical Form			
		Indeterminate	Cardiac	Digestive	Cardiodigestive
Parameters		n = 72	n = 74	n = 20	n = 27
Age (years)	Average ± SD	55.7 ± 8.3	59.2 ± 10.0	59.9 ± 11.6	57.8 ± 9.9
Gender (%)	Female	69.5	51.4	80.0	40.7
Ethnic group (%)	European	84.7	67.6	60.0	74.1
	African	11.1	25.7	35.0	22.2
	Asian	0	1.3	0	0
	Amerindian	4.2	5.4	5.0	3.7
Functional classification of cardiac insufficiency	A	2	16	n.a.	9
ACC/AHA^&^	B1	4	18	n.a.	5
	B2	0	3	n.a.	0
	C	2	26	n.a.	10
	D	0	2	n.a.	1
MBL levels [28]	Median (n):	1314 (47)	1441 (44)	1883 (12)	2119 (19)
(ng/ml)	[Min-Max]	[50–6379]	[50–7214]	[50–4700]	[50–5600]

“A” means altered electrocardiogram (ECG) and normal echocardiogram (ECO); “B1” means altered ECO, left ventricular ejection fraction (LVEF) higher than 45% and no cardiac insufficiency (CI); “B2” means altered ECO, LVEF lower than 45% and no CI; “C” means altered ECG and ECO and compensable CI; “D” means altered ECG and ECO and refractory CI.; n.a. = not applicable; SD = standard deviation; n = number of individuals

^&^ At the time of blood sampling, three patients were only defined as *T*. *cruzi-*infected, 64 patients with the indeterminate, 9 with the cardiac and two with the cardiodigestive form of the disease have not been graded for functional classification of the ACC/AHA.

### Classification of the cardiac patients

Stages of heart failure of the cardiac patients were determined according to the guidelines of the American College of Cardiology and American Heart Association (ACC/AHA), adapted for CD as suggested by the Brazilian Consensus on Chagas disease [32]. Using these criteria, we graded CD cardiac patients in five different classes, which allows a functional classification of cardiac insufficiency as well as the identification of distinct sub-groups for prognostic and clinical management, as follows: **A**: altered electrocardiogram (ECG) and normal echocardiogram (ECO); **B1**: altered ECO, left ventricular ejection fraction (LVEF) >45%, cardiac insufficiency (CI) absence; **B2**: altered ECO, LVEF <45%, CI absence; **C**: altered ECG and ECO, compensable CI; **D**: altered ECG and ECO, refractory CI. After signing the formal written consent, five ml of venous blood from each individual was collected and distributed in two collection tubes, one containing ethylenediamine tetra-acetic acid (EDTA) and other without the anticoagulant. Buffy coat and serum samples separation was performed as quickly as possible. Samples were kept on ice after being collected, during transport to the laboratory and separation, and immediately stored at -80°C until used. Genomic DNA was extracted from peripheral whole blood using commercial kits (GFX Genomic Blood DNA Purification Kit, GE Healthcare, São Paulo, Brazil), according to the manufacturer’s instructions.

### MBL measurement

We used previous published data on MBL serum levels [29] and on cytokine and chemokine levels [33] from the same patient cohort in order to evaluate if *MBL2* genotypes were associated with these phenotypes. *MBL2* genotypes were grouped according to the published influence of *X/Y* promoter and *A/B/C/D* (or *A/O*) exon 1 variants on MBL serum levels: *YA/YA* and *XA/YA* (high MBL concentration), *YA/YO* (intermediate MBL concentration), *XA/XA*, *XA/YO* and *YO/YO* (low MBL concentrations) [31,34].

### *MBL2* sequencing

A fragment of 1059 nucleotides was amplified using the forward primers MBL_PromF (5'-GCCAGAAAGTAGAGAGGTATTTAGCAC-3') and the reverse primer MBL_Rev (5'-CCAACACGTACCTGGTTCCC-3'). The PCR fragments were stained with SYBR Safe DNA Gel Stain (Invitrogen, Carlsbad, USA) and visualized on a 1% (w/v) agarose gel. The PCR products were purified using ExoSAP-It (GE Healthcare, Uppsala, Sweden). All fragments were sequenced with the amplification primers (IDT, Florida, USA) or an internal exon 1 sequencing primer, MBL_EX1F (5'- CAGGTGTCTAGGCACAGATGAACC-3'), using Big dye terminator version 1.1 chemistry (Applied Biosystems, CA, USA), precipitated using ammonium acetate (7,5M), denatured with formamide (Applied Biosystems) and analysed on an automated sequencer (ABI Prism 3500xL Genetic Analyzer, Applied Biosystems). Sequencing data was analyzed using Geneious v5.4 (Biomatters Ltd, Auckland, New Zealand) and SeqScape v2.7 (Applied Biosystems) softwares.

### Statistical analysis

Genotype and allele frequencies were obtained by direct counting. The hypothesis of Hardy–Weinberg equilibrium (based on the approach of Guo and Thompson) [35] was tested using ARLEQUIN software package version 3.5.1.3 (http://anthro.unige.ch/arlequin/). Possible associations between *MBL2* genotypes/haplotypes/alleles and different clinical forms were evaluated with two tailed Fisher’s exact test. Distribution of MBL and cytokine/chemokine concentrations according to *MBL2* genotypes in the different groups were compared using t-test or ANOVA and, if not normally distributed, with Mann-Whitney or Kruskal-Wallis tests. Unless otherwise stated, two-tailed P-values less than 5% were considered significant, presented “as is” and Bonferroni-corrected. These analyses were done using the Graphpad Prism 5.04 software package.

## Results

### *MBL2* polymorphisms

Genotype distribution was in Hardy and Weinberg equilibrium for all investigated SNPs in both patient and control groups. We identified eight *MBL2* haplotypes comprehending the *-221 (H/L)* and *-550 (X/Y)* promoter SNPs, *+4 (P/Q)* SNP in the 5’ untranslated region, codon 52 (*A/D*), codon 54 (*A/B*) and codon 57 (*A/C*) SNPs in exon 1 (Table 2). The uncommon *LYPD* haplotype occurred in a single patient presenting the associated clinical form of CD. The MBL2*C variant, imbedded in the *LYQC* haplotype, was negatively associated with the disease (7/392 or 1.8% in patients vs. 22/404 or 5.4% in controls, OR = 0.32 [95%CI = 0.13–0.75], P = 0.007, P_BF_ = 0.029). This effect was restricted to patients having either the cardiac, digestive or cardiodigestive forms of the disease (2/242 or 0.8% in symptomatic patients vs. 22/404 or 5.4% in controls, OR = 0.14 [95%CI = 0.03–0.62], P = 0.002, P_BF_ = 0.008). *LYQC* was absent from cardiac CD patients (compared with controls: P = 0.001, P_BF_ = 0.004; compared with indeterminate patients: P = 0.028, P_BF_ = 0.11).

**Table 2:** Distribution of *MBL2* alleles and haplotypes in patients and controls.

	Controls		Patients		Symptomatic *		Indeterminate		Cardiac		Digestive		Cardiodigestive	
*MBL2*	N = 404	%	N = 392	%	N = 242	%	N = 144	%	N = 148	%	N = 40	%	N = 54	%
Alleles														
*H>L*	286	70.8	256	65.3	159	65.7	95	66.0	97	65.5	27	67.5	35	64.8
*X>Y*	337	83.4	316	80.6	190	78.5	121	84.0	117	79.1	29	72.5	44	81.5
*P>Q*	111	27.5	91	23.2	55	22.7	35	24.3	34	23.0	9	22.5	12	22.2
*A>D*	11	2.7	16	4.1	10	4.1	5	3.5	5	3.4	2	5.0	3	5.6
*A>B*	63	15.6	51	13.0	29	12.0	22	15.3	18	12.2	5	12.5	6	11.1
***A>C***	**22**	**5.4**	**7**	**1.8**	**2**	**0.8**	**5**	**3.5**	**0**	**0**	**1**	**2.5**	**1**	**1.9**
Haplotypes														
*HYPA*	107	26.5	121	30.9	74	30.6	44	30.6	46	31.1	11	27.5	17	31.5
*HYPD*	11	2.7	15	3.8	9	3.7	5	3.5	5	3.4	2	5.0	2	3.7
*LYPA*	45	11.1	37	9.4	22	9.1	15	10.4	14	9.5	2	5.0	6	11.1
*LYPB*	63	15.6	51	13.0	29	12.0	22	15.3	18	12.2	5	12.5	6	11.1
*LYQA*	89	22.0	84	21.4	53	21.9	30	20.8	34	23.0	8	20.0	11	20.4
*LXPA*	67	16.6	76	19.4	52	21.5	23	16.0	31	20.9	11	27.5	10	18.5
***LYQC***	**22**	**5.4**	**7**	**1.8**	**2**	**0.8**	**5**	**3.5**	**0**	**0**	**1**	**2.5**	**1**	**1.9**
*LYPD*	0	0	1	0.25	1	0.4	0	0	0	0	0	0	1	1.9

Allele frequencies are given for the second variant, e.g. for *L* in the case of *H>L*. With **Y16577** as Genbank reference sequence, *H>L* is *g*.*273G>C* (rs11003125), *X>Y* is *g*.*602G>C* (rs7096206), *P>Q* is *g*.*826C>T* (rs7095891), *A>D* is *g*.*1045C>T* (rs5030737), *A>B* is *g*.*1052G>A* (rs1800450) and *A>C* is *g*.*1061G>A* (rs1800451). Haplotypes could be deduced due to the strong linkage disequilibrium between the alleles [48]. In the haplotypes, the *A>D*, *A>B* and *A>C* alleles were considered as only one locus, due to their close proximity. N = number of chromosomes. In bold: allele *C* and haplotype *LYQC* negatively associated with the disease, whose frequencies differed between patients and controls (P = 0.007, P_BF_ = 0.029), symptomatic patients and controls (P = 0.002, P_BF_ = 0.008), cardiac patients and controls (P = 0.001, P_BF_ = 0.004), cardiac and indeterminate patients (P = 0.028, P_BF_ = 0.11).

* Symptomatic patients include cardiac, digestive and cardiodigestive forms.

**Fig 1:**
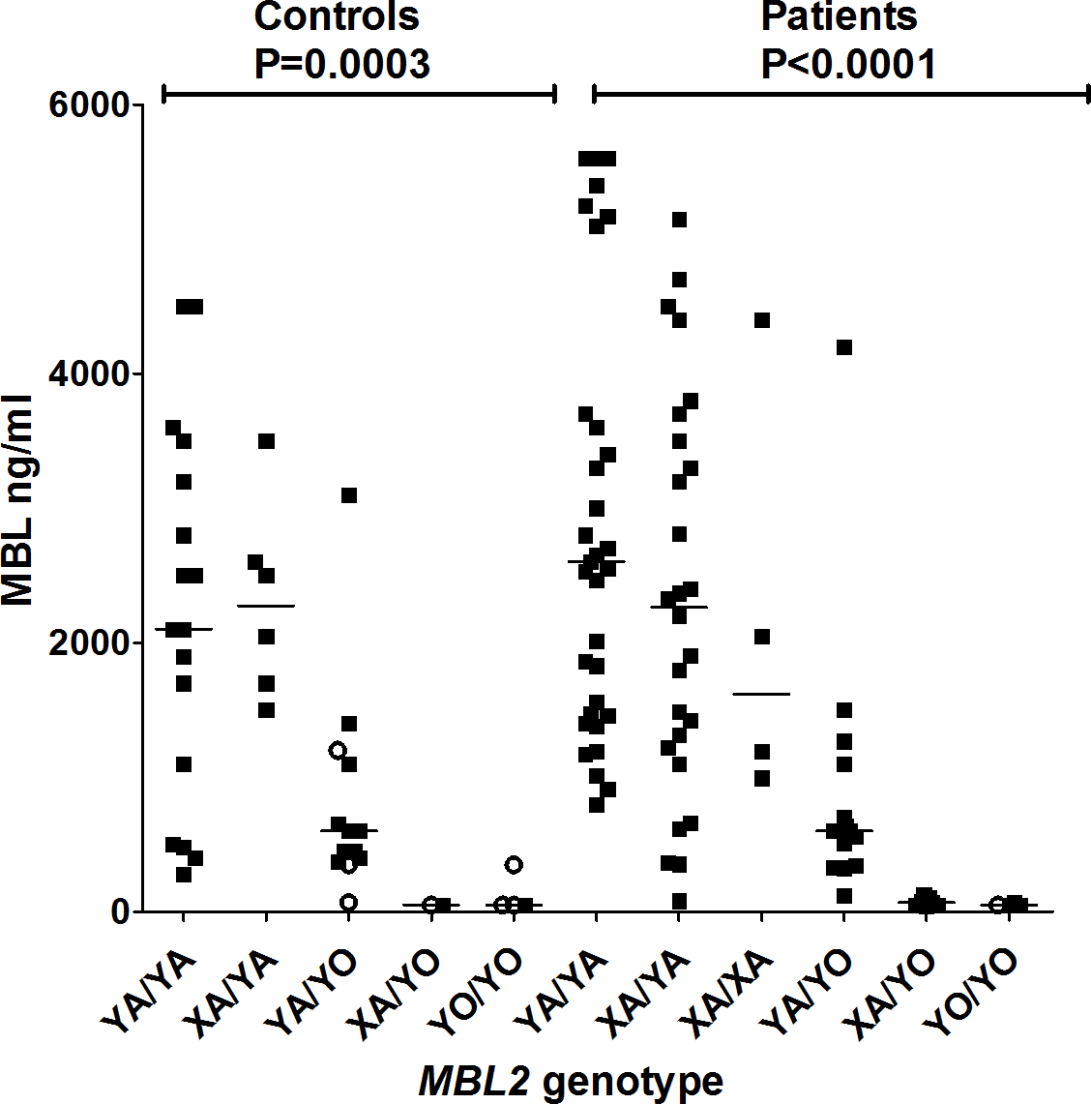
Distribution of MBL levels according to *MBL2* genotypes in controls and patients. Open circles indicate individuals with the *LYQC* haplotype. Medians in each group are given by a horizontal line. P values refer to Kruskal-Wallis test.

There was no particular genotype with the *C* allele (in the *LYQC* haplotype), associated with the disease, but summed genotype frequencies with this allele/haplotype did differ between symptomatic patients and controls (2/121 or 1.7% vs. 19/202 or 9.4%, respectively, OR = 0.16 [95%CI = 0.04–0.71], P = 0.005, P_BF_ = 0.02), especially between cardiac patients and controls (0/74 vs. 19/202 or 9.4%, P = 0.003; P_BF_ = 0.012). In fact, *YC/YC* and *YA/YC* genotypes were not identified in symptomatic patients (S1 Table). As previously published [29], we did not find a difference between MBL levels in controls and patient groups (S1 Fig), but confirmed the well established association between the *X/Y* and *A/O* variants and the MBL levels in both patients and controls (Fig 1).

### *MBL2 g*enotypes and functional classification of heart failure

**Fig 2:**
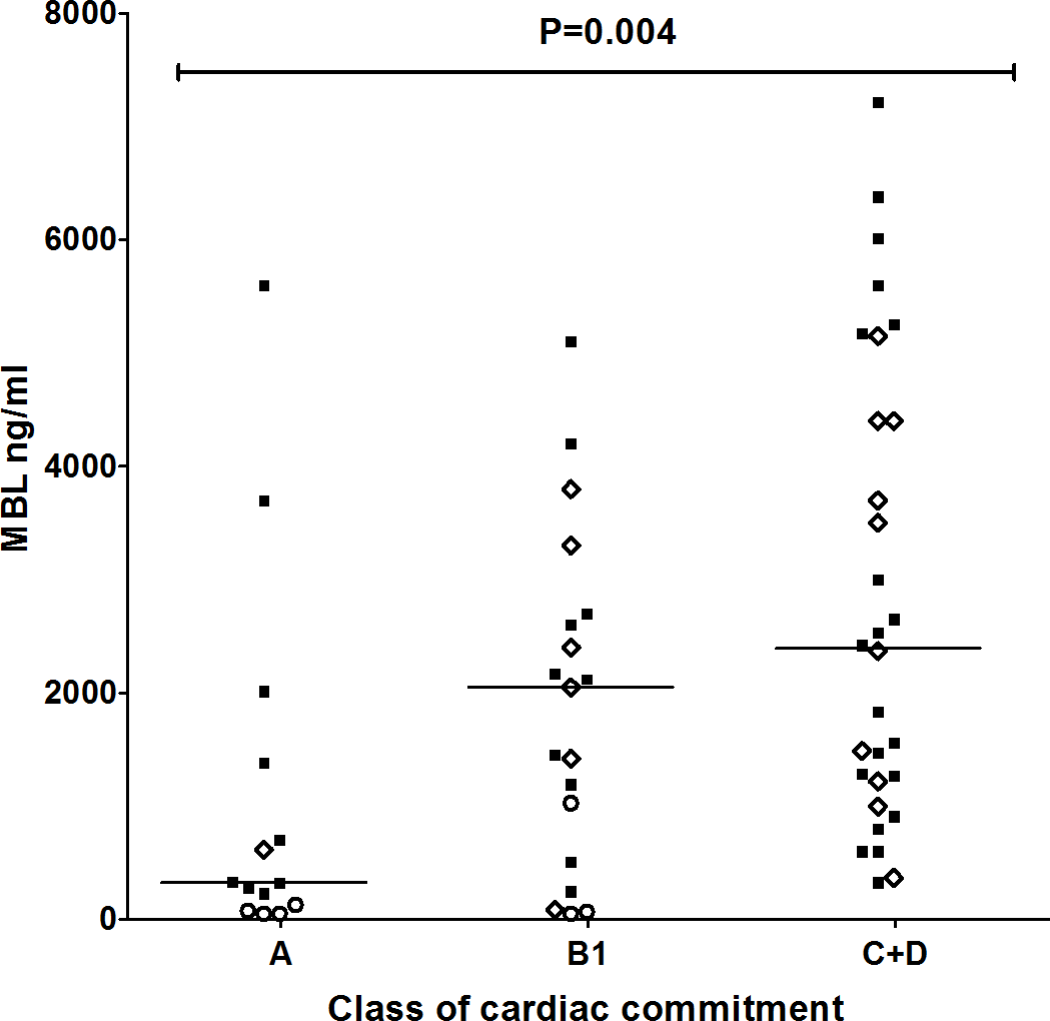
Distribution of MBL levels according to the functional classification of heart failure. Open circles indicate patients with the *YO* haplotype; open diamonds, patients with the *XA/XA* or *XA/YA* genotypes. Medians in each group are given by a horizontal line. MBL levels were not analyzed in patients classified within the “B2” class. P value refers to Kruskal-Wallis test.

Genotypes with the *YO* haplotype (*YA/YO*, *XA/YO* and *YO/YO*) were more common in patients without echocardiographic alterations (15/28 or 53.6% in patients classified within the “A” class vs. 15/71 or 21.1% in patients classified within “B”, “C” or “D” classes, OR = 0.23 [95%CI = 0.09–0.59], P = 0.003, P_BF_ = 0.009). Fourteen out of 17 individuals with the *XA/YO and YO/YO* genotypes, including patients and controls (82.4%), presented MBL deficiency (MBL levels lower than 100 ng/ml), being three in the A group, three in B1 and none in the C and D groups (Fig 2). In contrast, individuals with the *XA/XA* and *XA/YA* genotypes were more frequent in the patients with echocardiographic alterations (2/28 or 7.1% in the “A” class vs. 25/71 or 35.2% in the “B+C+D” class, OR = 7.07 [95%CI = 1.55–32.2], P = 0.005, P_BF_ = 0.015) (Table 3). Thirteen out of 17 individuals with these genotypes presented MBL levels higher than 1000 ng/ml (Fig 2).

**Table 3:** *MBL2* genotype distribution according to functional classification of heart failure.

Class	A		B1 + B2 *		C + D *		B + C + D	
*MBL2*	n = 28	%	n = 30	%	n = 41	%	n = 71	%
*YA/YA*	11	39.3	12	40.0	19	46.3	31	43.7
***XA/XA***	**0**	**0**	2	6.7	3	7.3	**5**	**7.0**
***XA/YA***	**2**	**7.1**	8	26.7	12	29.3	**20**	**28.2**
***YA/YO***	**9**	**32.1**	5	16.7	7	17.1	**12**	**16.9**
***XA/YO***	**3**	**10.7**	2	6.7	0	0.0	**2**	**2.8**
***YO/YO***	**3**	**10.7**	1	3.3	0	0.0	**1**	**1.4**

In bold, genotypes who’s summed frequencies (*XA/XA* + *XA/YA* and *YA/YO* + *XA/YO* + *YO/YO*) did differ between patients classified in the A and in the joined B+C+D groups. “A” means altered electrocardiogram (ECG) and normal echocardiogram (ECO); “B1” means altered ECO, left ventricular ejection fraction (LVEF) higher than 45% and no cardiac insufficiency (CI); “B2” means altered ECO, LVEF lower than 45% and no CI; “C” means altered ECG and ECO and compensable CI; “D” means altered ECG and ECO and refractory CI.

* There were only 3 patients classified in the B2 class, as well as in the D class.

n = number of individuals.

### *MBL2* genotypes and cytokine/chemokine levels

**Fig 3:**
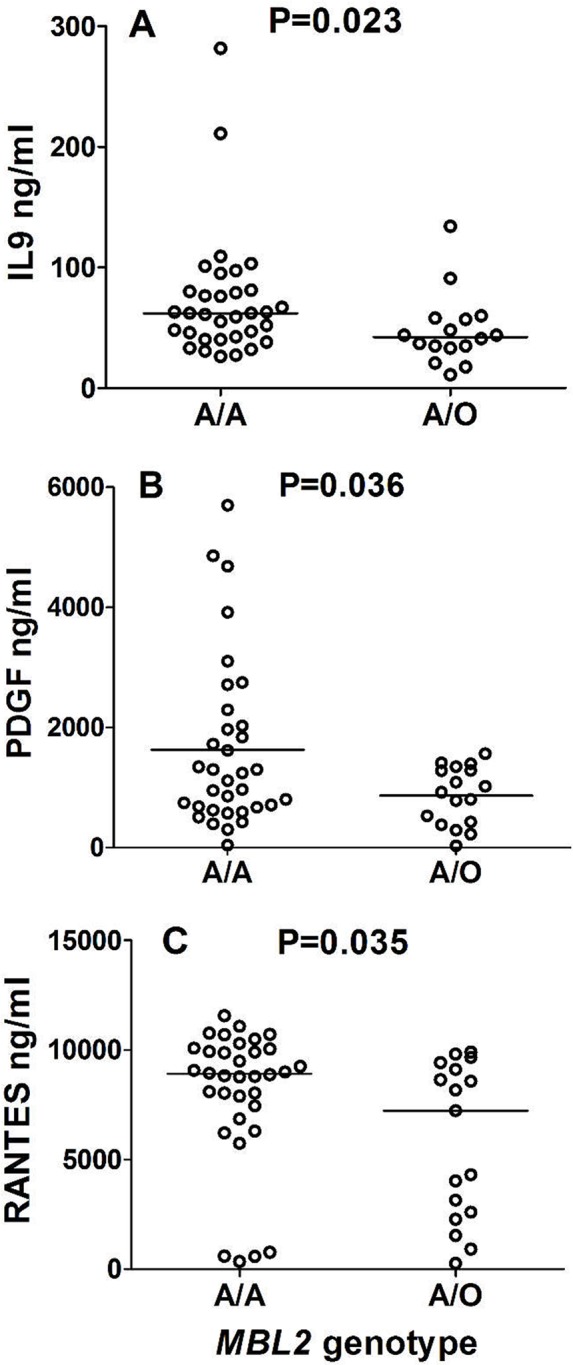
Distribution of cytokine/chemokine levels according to the presence of MBL2*O (*B*, *C* or *D*) alleles. A. IL9 distribution (P value refers to Mann-Whitney test; horizontal line indicates the median level). B. PDGF distribution (P value refers to an unpaired t-test; horizontal line indicates the mean level). C. RANTES distribution (P value refers to Mann-Whitney test; horizontal line indicates the median level). There were no MBL2*O/O homozygotes among those measured for the investigated cytokines/chemokines. Three outliers with inconsistent results were excluded from all comparisons. Due to small sample size, Bonferroni P values were not significant.

We found a trend for lower levels of pro-inflammatory interleukin 9 (IL-9), platelet-derived growth factor (PDGF) and Regulated-on-activation, T-cell expressed and secreted (RANTES, renamed CCL5) in patients with *O* variants (Fig 3). No significant associations were observed for previously investigated levels of interleukin 1 receptor antagonist (IL-1RA), IL-17 and interferon gamma (IFN-γ), eotaxin and granulocyte-colony stimulating factor (G-CSF) in the same individuals.

## Discussion

The process of opsonization and phagocytosis of parasites and its destruction or survival within phagocytic cells is crucial for the establishment of most infectious diseases. It is known that MBL plays a central role in the initial interaction between pathogens and phagocytes, mediating opsonization and phagocytosis, either directly or by activating the antibody-independent lectin pathway of the complement system [36]. Importantly, complement is one of the first lines of immune defense to interact with infective forms of *T*. *cruzi*, long before the development of antibodies. In fact, MBL is able to bind to infective forms of *T*. *cruzi* [15], acting directly in the control of parasitemia [37]. The major surface glycoprotein of *T*. *cruzi* amastigotes, named SA85-1, is a ligand for human MBL and adhesion of amastigotes to macrophages is facilitated by mannose receptor [38,39]. In addition, mannose receptors on cardiomyocytes where shown to be involved in the binding and internalization of *T*. *cruzi* [40,41]. Recently, a role for MBL in the regulation of host resistance on myocardial inflammation has been described in *T*. *cruzi* experimental infection [16].

To our knowledge, this is the first work to investigate *MBL2* polymorphisms in Brazilian patients with CD and to suggest a role for MBL in chronic disease. We found that MBL*2 C allele, imbedded in the *LYQC* haplotype and conferring MBL deficiency, is associated with protection against the development of symptomatic forms of CD. Interestingly, a protective effect could be extended for all deficiency-associated exon 1 variants (*B*, *C* and *D*, also called *O*) against the myocardial injury, which could lead to development of echocardiographic alterations, corroborating the hypothesis that MBL deficiency may protect against the progression to severe CCC. On the other hand, higher frequency of *XA/YA* and *XA/XA* genotypes in patients with echocardiographic alterations partly explains higher MBL levels in the patient group graded within the B, C and D stages of functional classification, as previously published [29]. One must consider that other factors may affect MBL production, such as growth [42], thyroid hormones [43] and the acute phase response [44]. However, it is important to note that these physiological variables increase MBL levels by no more than three times, thus genetic variation in the *MBL2* gene seems to most significantly influence MBL protein levels in the disease [26].

We were unable to directly compare these results with our former work done on C3 and BF allotypes [12], since samples were not the same. Nevertheless the functional impact of the C3F allele has been recently demonstrated [45]: the p.Arg102Gly amino acid substitution responsible for the “fast” electrophoretic mobility of the C3F allele disrupts a salt bridge necessary for stable interaction with factor H, causing deregulated activation of the alternative pathway. Not surprisingly, this allele increases inflammatory activity and was found associated with the cardiac form of CD [12].

Corroborating our association of the MBL2*LYQC haplotype as well as *YA/YO* and *YO/YO* genotypes with the indeterminate, or asymptomatic, clinical form in *T*. *cruzi* infected individuals, all three have been associated with positive parasitemia counts in asymptomatic adults infected with *Plasmodium falciparum* [46]. A similar association was found with protection against the lepromatous manifestation of leprosy, compared with the less severe tuberculoid form [22]. On the other hand, the MBL2*B allele, imbedded in the *LYPB* haplotype and also associated with MBL deficiency, was more frequent in CD patients than in healthy individuals from northern Chile, but did not differ between asymptomatic and CCC patients. Noteworthy, MBL2*C and MBL2*D alleles were not observed in Chilean patients or controls [47]. It is important to consider that *MBL2* allele frequencies differ greatly between populations [48,49] being 42–46% for MBL2*B in Guarani Amerindians from south Brazil [31] and Chiriguano and Mapuche Indians from Argentina [50], and 11% for Euro-Brazilians from South Brazil [31], as those investigated in the present study. Thus, the high frequency of MBL2*B found in Chilean patients (48%) is probably due to high Amerindian admixture (52%, based on ancestry-informative markers [51]. The frequency of this allele was much lower in the present study (13% in patients and 15.6% in controls), in accordance with the very low Amerindian admixture (5%) of the investigated population [52,53]. Beside these differences in population structure, functional differences among the MBL2*B, *C* and *D* variants regarding MASP-2 coupling [54] and serum concentration of low-mass oligomers [55] may explain opposite association outcomes and should be further investigated in the context of Chagas disease [31].

In the last decade, cumulative evidence has pointed to an immunopathological role for MBL in both experimental as well as clinical studies of cardiac disorders. High levels of MBL were associated, for example, with increased risk of ischemia, myocardial infarction and sudden death in patients with rheumatoid arthritis. A critical role for MBL in ischemia and reperfusion injury of the ischemic myocardium in experimental diabetes has been shown [23], with MBL enhancing post-ischemic reperfusion injury and its deficiency protecting against this damage [56]. In fact, MBL deficiency was suggested to reduce tissue damage, arrhythmias and mortality of patients after myocardial infarction [57], probably due to endothelial cell binding, followed by excessive complement activation [19]. In addition, *MBL2* genotypes associated with high MBL levels were shown to increase the risk of acute and chronic carditis in patients with rheumatic fever, whereas *O* alleles were protective [24,58]. In the same way, despite the beneficial role of complement in early infection to control the parasite load, an excessive activation of complement during the chronic phase of CD can be damaging to the host, contributing to tissue damage and injury of the affected organs. It was previously reported that the persistence of the parasite in the chronic stage could cause desialylation of myocardial and endothelial cells, leading to complement activation and deposition of the membrane attack complex on cellular surfaces [11].

Furthermore, we found an association of higher pro-inflammatory cytokine IL-9 and PDGF, as well as of the chemokine RANTES, in patients with the MBL2*A/A genotype. This effect could be dependent on multimeric MBL forms, known to regulate the release of different cytokines from monocytes and other immune cells in response to infection [31,59]. Although IL-9 was not yet implicated in CD, PDGF was associated with proliferative lesions and fibrosis in CCC [60] and RANTES is among the highest expressed genes in dogs with intense cardiac parasitism and in end-stage CCC patients [61,62]. All these findings allow speculation for the use of inhibitors of the lectin pathway, as a preventive therapy to reduce tissue injury in inflammatory cardiac disorders and other chronic inflammatory diseases where activation of the lectin pathway takes place [63].

**Fig 4:**
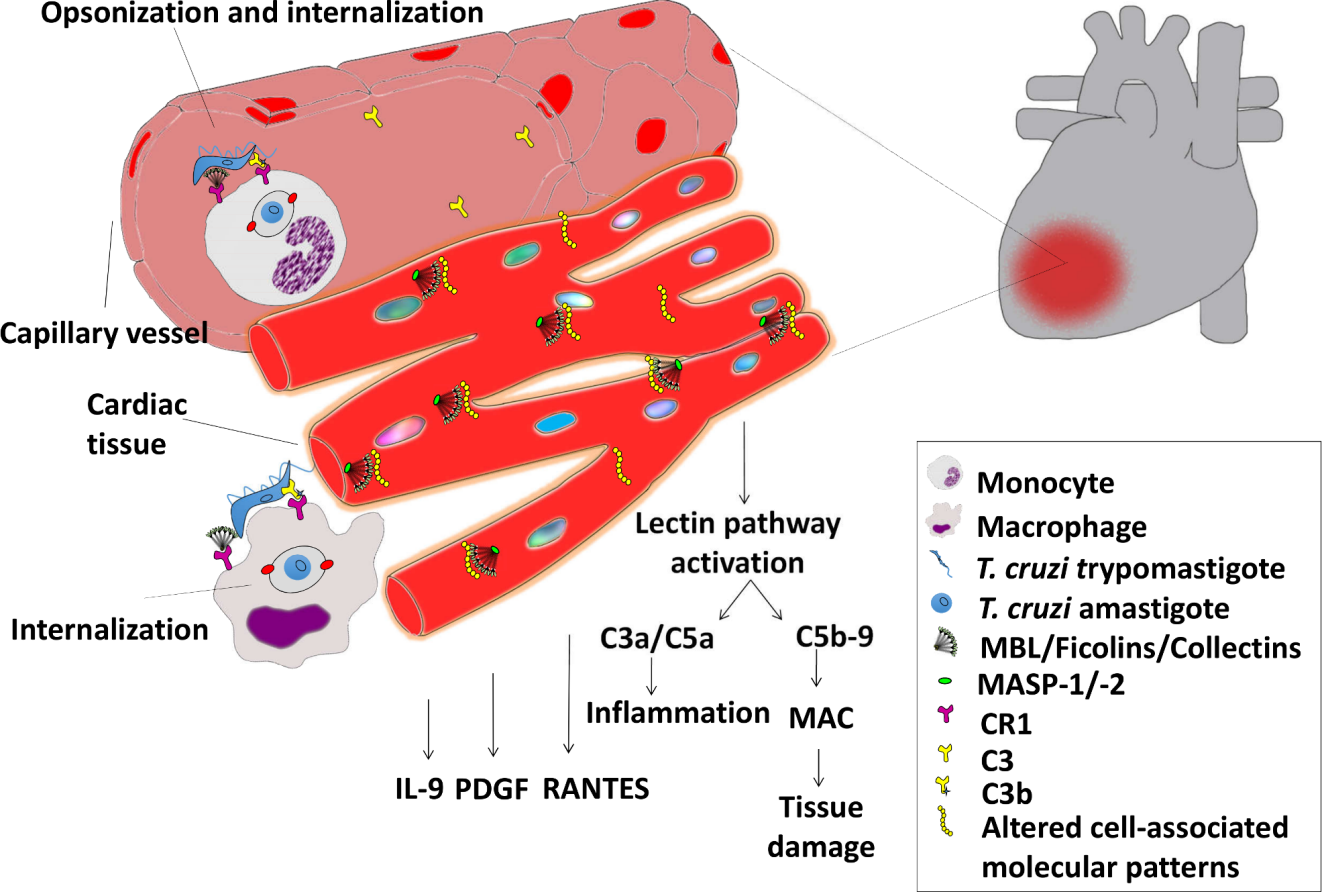
Hypothetical role of high MBL levels in heart Chagas disease. In the acute stage of *T*. *cruzi* infection, MBL molecules function as opsonins for the pathogen. Thus high MBL levels would increase phagocytosis of the parasite. In the chronic stage of the disease, MBL may bind to altered-cell molecular patterns expressed on myocardium of CD patients, activating the lectin pathway and leading to an increased secretion of RANTES and pro-inflammatory cytokines such as IL-9 and PDGF (in patients with the MBL2*A/A genotype), thereby promoting heart damage leading to chagasic chronic cardiomiopathy.

Our hypothesis is that high levels of MBL, which are genetically determined, could facilitate the internalization of *T*. *cruzi* in macrophages and cardiomyocytes thereby increasing cellular invasion by the parasite and its consequent dissemination to target organs, increasing complement-mediated tissue injury and cardiac damage in the chronic stage of the disease (Fig 4).

MBL could bind to the myocardium of CD patients expressing: 1)*T*. *cruzi* antigens, 2) self-antigens from the host presenting molecular mimicry with parasite epitopes or 3) neoantigens containing MBL ligands, exposed after tissue injury. Due to high levels, MBL could deposit and overly activate the lectin pathway, corroborating to persistent inflammation and tissue damage, reparative fibrosis and cardiac dysfunction. In fact, increased deposition of complement terminal lytic complex in the myocardium of patients with CCC, suggested an association of complement activation with active inflammation and fibrosis in CCC [11]. Thus, despite of the important role of complement in controlling the initial *T*. *cruzi* infection and parasite replication, CD patients with *MBL2* genotypes conferring high production of MBL seem to be prone to develop cardiac dysfunction probably due to excessive complement activation. Similarly, MBL deficiency could protect against cellular invasion by *T*. *cruzi* and minimize the exacerbated damage caused by unwarranted activation of complement. Thus, quantification of serum MBL and *MBL2* genotyping might be useful markers for prognostic and clinical evolution of CD, especially of CCC. However, additional studies are needed in order to replicate these findings and to confirm this hypothesis.

## Supporting Information

S1 Fig Distribution of MBL levels in controls and patient groups. Note: Black circles indicate individuals with the *LYQC* haplotype. Medians in each group are given by a horizontal line.
(TIF)

S1 Table Distribution of *MBL2* genotypes in Chagas patients and controls. In bold: genotypes with the *C* variant, whose summed frequencies differ between patients and controls (P = 0.024, P_BF_ = 0.096), symptomatic patients and controls (P = 0.005, P_BF_ = 0.020), cardiac patients and controls (P = 0.003, P_BF_ = 0.012), cardiac and indeterminate patients (P = 0.027, P_BF_ = 0.108) (see text). * Symptomatic patients include cardiac, digestive and cardiodigestive forms. n = number of individuals.
(DOCX)
