# Supplementary figures and images for: Genetically Determined MBL Deficiency Is Associated with Protection against Chronic Cardiomyopathy in Chagas Disease
Source: PLoS Negl Trop Dis. 2016 Jan 8;10(1):e0004257. doi: 10.1371/journal.pntd.0004257 (PMC4706301; doi:10.1371/journal.pntd.0004257)

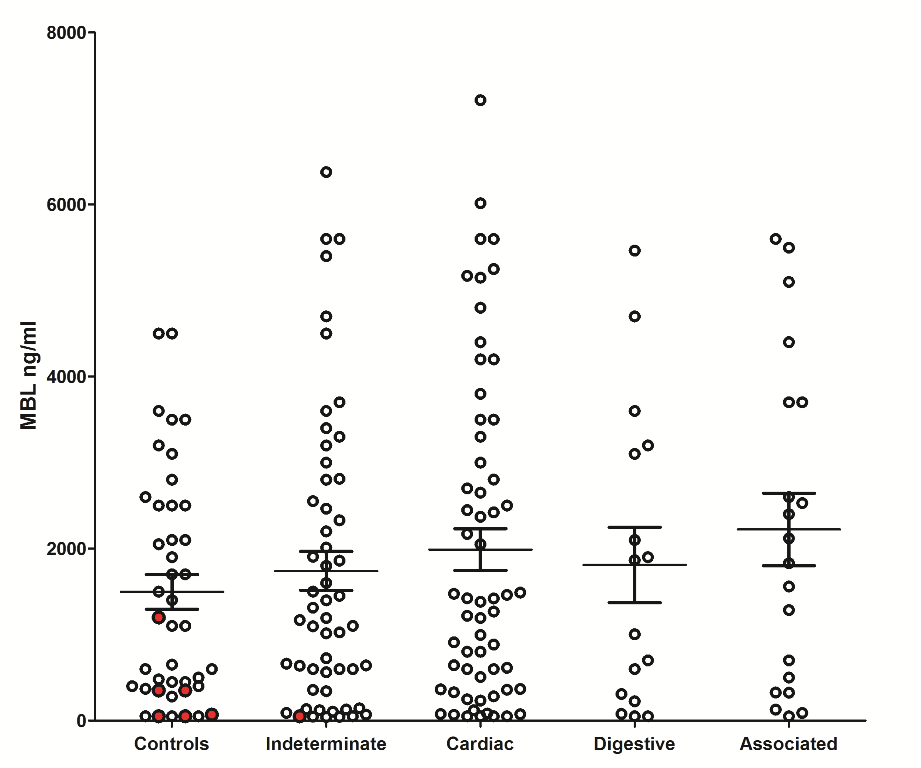

Supplement: S1 Fig — Note: Black circles indicate individuals with the LYQC haplotype. Medians in each group are given by a horizontal line. (TIF) [file pntd.0004257.s001.tif]
