# Supplementary material for: Genetically Determined MBL Deficiency Is Associated with Protection against Chronic Cardiomyopathy in Chagas Disease
Source: PLoS Negl Trop Dis. 2016 Jan 8;10(1):e0004257. doi: 10.1371/journal.pntd.0004257 (PMC4706301; doi:10.1371/journal.pntd.0004257)
Supplement: S1 Table — In bold: genotypes with the C variant, whose summed frequencies differ between patients and controls (P = 0.024, PBF = 0.096), symptomatic patients and controls (P = 0.005, PBF = 0.020), cardiac patients and controls (P = 0.003, PBF = 0.012), cardiac and indeterminate patients (P = 0.027, PBF = 0.108) (see text). * Symptomatic patients include cardiac, digestive and cardiodigestive forms. n = number of individuals. (DOCX) [file pntd.0004257.s002.docx]

|  | Controls | | Patients | | Symptomatic * | | Indeterminate | | Cardiac | | Digestive | | Cardiodigestive | |
| --- | --- | --- | --- | --- | --- | --- | --- | --- | --- | --- | --- | --- | --- | --- |
| Genotypes | n=202 | % | n=196 | % | n=121 | % | n=72 | % | n=74 | % | n=20 | % | n=27 | % |
| *HYPA/HYPA* | 21 | 10.40 | 22 | 11.22 | 14 | 11.57 | 7 | 9.72 | 8 | 10.81 | 2 | 0.1 | 4 | 14.81 |
| *HYPA/HYPD* | 2 | 1.00 | 5 | 2.55 | 3 | 2.48 | 2 | 2.78 | 2 | 2.70 | 0 | 0 | 1 | 3.70 |
| *HYPA/LYPA* | 11 | 5.45 | 11 | 5.61 | 6 | 4.96 | 5 | 6.94 | 4 | 5.41 | 1 | 0.05 | 1 | 3.70 |
| *HYPA/LYPB* | 21 | 10.40 | 15 | 7.65 | 10 | 8.26 | 5 | 6.94 | 5 | 6.76 | 2 | 0.1 | 3 | 11.11 |
| *HYPA/LYQA* | 21 | 10.40 | 20 | 10.20 | 11 | 9.09 | 9 | 12.50 | 8 | 10.81 | 1 | 0.05 | 2 | 7.41 |
| *HYPD/LYPB* | 2 | 1.00 | 1 | 0.51 | 1 | 0.83 | 0 | 0.00 | 1 | 1.35 | 0 | 0 | 0 | 0.00 |
| *HYPD/LYQA* | 4 | 2.00 | 6 | 3.06 | 3 | 2.48 | 2 | 2.78 | 2 | 2.70 | 1 | 0.05 | 0 | 0.00 |
| *HYPA/LXPA* | 8 | 4.00 | 26 | 13.27 | 16 | 13.22 | 9 | 12.50 | 11 | 14.86 | 3 | 0.15 | 2 | 7.41 |
| *HYPD/LXPA* | 2 | 1.00 | 3 | 1.53 | 2 | 1.65 | 1 | 1.39 | 0 | 0.00 | 1 | 0.05 | 1 | 3.70 |
| *LXPA/LXPA* | 8 | 4.00 | 8 | 4.08 | 7 | 5.79 | 1 | 1.39 | 4 | 5.41 | 1 | 0.05 | 2 | 7.41 |
| *LXPA/LYPA* | 8 | 4.00 | 10 | 5.10 | 6 | 4.96 | 4 | 5.56 | 4 | 5.41 | 1 | 0.05 | 1 | 3.70 |
| *LXPA/LYPB* | 11 | 5.45 | 9 | 4.59 | 5 | 4.13 | 4 | 5.56 | 3 | 4.05 | 2 | 0.1 | 0 | 0.00 |
| *LXPA/LYQA* | 17 | 8.42 | 10 | 5.10 | 7 | 5.79 | 3 | 4.17 | 5 | 6.76 | 1 | 0.05 | 1 | 3.70 |
| ***LXPA/LYQC*** | **5** | **2.48** | **2** | **1.02** | **2** | **1.65** | **0** | **0.00** | **0** | **0.00** | 1 | 0.05 | 1 | 3.70 |
| *LYPA/LYPA* | 5 | 2.48 | 1 | 0.51 | 0 | 0.00 | 1 | 1.39 | 0 | 0.00 | 0 | 0 | 0 | 0.00 |
| *LYPA/LYPB* | 1 | 0.50 | 3 | 1.53 | 1 | 0.83 | 2 | 2.78 | 1 | 1.35 | 0 | 0 | 0 | 0.00 |
| *LYPA/LYQA* | 12 | 5.94 | 9 | 4.59 | 9 | 7.44 | 0 | 0.00 | 5 | 6.76 | 0 | 0 | 4 | 14.81 |
| ***LYPA/LYQC*** | **2** | **1.00** | **2** | **1.02** | **0** | **0.00** | **2** | **2.78** | **0** | **0.00** | 0 | 0 | 0 | 0.00 |
| *LYPB/LYPB* | 5 | 2.48 | 2 | 1.02 | 1 | 0.83 | 1 | 1.39 | 1 | 1.35 | 0 | 0 | 0 | 0.00 |
| *LYPB/LYPD* | 0 | 0.00 | 1 | 0.51 | 1 | 0.83 | 0 | 0.00 | 0 | 0.00 | 0 | 0 | 1 | 3.70 |
| *LYPB/LYQA* | 13 | 6.44 | 17 | 8.67 | 9 | 7.44 | 8 | 11.11 | 6 | 8.11 | 1 | 0.05 | 2 | 7.41 |
| ***LYPB/LYQC*** | **5** | **2.48** | **1** | **0.51** | **0** | **0.00** | **1** | **1.39** | **0** | **0.00** | 0 | 0 | 0 | 0.00 |
| *LYQA/LYQA* | 10 | 5.00 | 10 | 5.10 | 7 | 5.79 | 3 | 4.17 | 4 | 5.41 | 2 | 0.1 | 1 | 3.70 |
| ***LYQA/LYQC*** | **2** | **1.00** | **2** | **1.02** | **0** | **0.00** | **2** | **2.78** | **0** | **0.00** | 0 | 0 | 0 | 0.00 |
| ***LYQC/LYQC*** | **3** | **1.49** | **0** | **0.00** | **0** | **0.00** | **0** | **0.00** | **0** | **0.00** | 0 | 0 | 0 | 0.00 |
| ***HYPA/LYQC*** | **2** | **1.00** | **0** | **0.00** | **0** | **0.00** | **0** | **0.00** | **0** | **0.00** | 0 | 0 | 0 | 0.00 |
| *HYPD/LYPA* | 1 | 0.50 | 0 | 0.00 | 0 | 0.00 | 0 | 0.00 | 0 | 0.00 | 0 | 0 | 0 | 0.00 |
